# Successful treatment of postoperative nonobstructive recurrent cholangitis by tract conversion surgery after total pancreatectomy: a case report

**DOI:** 10.1186/s40792-023-01686-9

**Published:** 2023-06-07

**Authors:** Masanori Odaira, Fumiki Toriumi, Shota Hoshino, Nozomi Iwama, Yasuhiro Ito, Takashi Endo, Hirohisa Harada

**Affiliations:** Department of Surgery, Tokyo Saiseikai Central Hospital, 1-4-17 Mita, Minato-Ku, Tokyo 108-0073 Japan

**Keywords:** Postoperative cholangitis, Biliary reconstruction, Hepaticojejunostomy, Afferent loop, Nonobstructive, Surgery, Complication

## Abstract

**Background:**

Postoperative cholangitis is a complication of biliary reconstruction during hepatobiliary pancreatic surgery. Most cases are associated with anastomotic stenosis, but there are also cases of cholangitis without stenosis, and treatment can be difficult, especially in patients with recurrent symptoms. In this report, we describe a case of repeated nonobstructive cholangitis in a patient after total pancreatectomy, in which a good outcome was obtained after performing tract conversion surgery.

**Case presentation:**

The patient was a 75-year-old man. He underwent total pancreatectomy for stage IIA cancer of the pancreatic body, hepaticojejunostomy via the posterior colonic route, gastrojejunostomy and Braun anastomosis via the anterior colonic route using the Billroth II method. The patient had a good postoperative course and was receiving adjuvant chemotherapy on an outpatient basis, but he developed his first episode of cholangitis 4 months after surgery. Although conservative treatment with antimicrobial agents was successful, the patient continued to have recurrent biliary cholangitis and was repeatedly admitted and discharged from the hospital. Since stenosis at the anastomosis was suspected, endoscopic observation of the anastomosis was performed using small bowel endoscopy for close examination, but no apparent stenosis was observed. Small bowel imaging indicated a possible influx of contrast medium into the bile duct, and reflux due to food residue was suspected as the cause of cholangitis. Since conservative treatment alone did not suppress the flare-up of symptoms, the decision was made to perform tract conversion surgery for curative purposes. The afferent loop was cut midstream, and jejunojejunostomy was performed downstream. The postoperative course was good, and the patient was discharged on the 10th day after surgery. He is currently an outpatient and has been free of cholangitis symptoms for 4 years without cancer recurrence.

**Conclusions:**

Although the diagnosis of nonobstructive retrograde cholangitis can be difficult, surgical treatment should be considered in patients with recurrent symptoms and refractory treatment.

## Background

Pancreatectomy is sometimes associated with serious short-term complications, but the number of such complications has decreased due to recent improvements in surgical and interventional techniques. On the other hand, with the improved outcomes of chemotherapy for malignant disease and the increased opportunities for surgery for low-grade disease, as typified by intraductal papillary mucinous neoplasm, there are more opportunities to address long-term complications after pancreatectomy. Long-term complications after pancreatic surgery include fatty liver, digestive failure, and diabetes mellitus [1]; postoperative cholangitis is a troublesome complication that affects a patient’s quality of life and is sometimes life-threatening [2]. Postoperative cholangitis is a challenging long-term complication not only in patients who undergo pancreatic surgery but also in those who undergo other hepatobiliary procedures involving biliary reconstruction (liver transplantation, hepatectomy, biliary resection) [3–5]. The incidence of cholangitis after biliary reconstruction is reported to occur in 5.7–18.6% of patients, which has a significant impact [2, 6–9]. If cholangitis is caused by anastomotic stenosis, endoscopic, percutaneous, or surgical treatment can improve the condition in most cases; however, if anastomotic stenosis is not the cause of cholangitis, antimicrobial therapy is usually the symptomatic treatment of choice, but no curative treatment has been established, especially in cases of recurrent symptoms.

This report describes a case in which a patient with repeated postoperative nonobstructive cholangitis underwent surgery and had a favorable outcome.

## Case presentation

The patient was a 75-year-old man. He underwent total pancreatectomy for stage IIA pancreatic body cancer, posterior colonic route hepaticojejunostomy, anterior colonic route Billroth II gastrojejunostomy, and Braun anastomosis. A 3-cm-diameter tumor was located in the pancreatic head. Intraoperative ultrasonography showed dilatation of the main pancreatic duct at the pancreatic tail and a nodule in the pancreatic duct, so intraductal papillary mucinous carcinoma coexistence could not be ruled out, and total pancreatectomy was selected for treatment. The operating time was 443 min, and the amount of blood loss was 250 ml. The patient had a good postoperative course and was discharged on the 18th day after surgery without complications. After discharge, the patient was started on adjuvant chemotherapy with S-1 for 6 months at the oncology outpatient clinic. After the start of chemotherapy, the patient had no major adverse events. Approximately 4 months after surgery, the patient presented to the emergency department with a chief complaint of fever. A CT scan showed no obvious findings other than pneumobilia, and blood tests showed an elevated inflammatory response and mild elevation of hepatobiliary enzymes. The patient was diagnosed with cholangitis and was hospitalized and treated with antimicrobial agents. The fever resolved promptly after the initiation of treatment. The blood culture was positive, and E. coli was detected. The patient was discharged after 2 weeks of antimicrobial therapy. After discharge from the hospital, chemotherapy was resumed, but the patient continued to have frequent episodes of fever, and although conservative treatment with antimicrobial agents was successful each time, the patient was repeatedly admitted to the hospital. The patient required a total of 16 hospitalizations and treatments over a 22-month period; blood cultures were positive in 11 of the 16 hospitalizations. Although the patient was managed in the oncology department, recurrent cholangitis made it difficult to continue chemotherapy; therefore, the patient was referred to the surgery department for consultation regarding treatment for recurrent cholangitis.

**Fig. 1:**
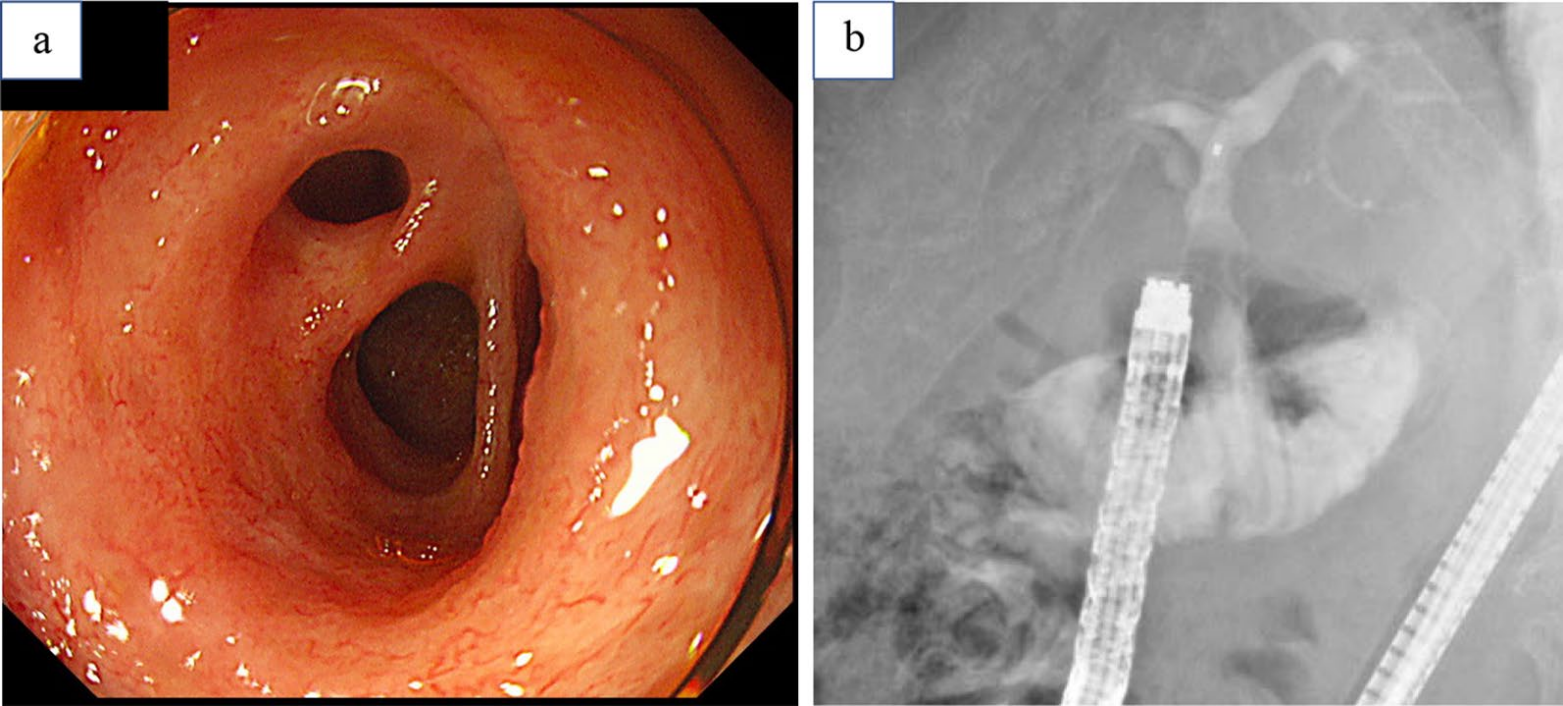
Small bowel endoscopic observation of the bile duct jejunal anastomosis showed no obvious stenosis (**a**). Fluoroscopic endoscopy showed no stenosis at the bile duct jejunal anastomosis (**b**)

**Fig. 2:**
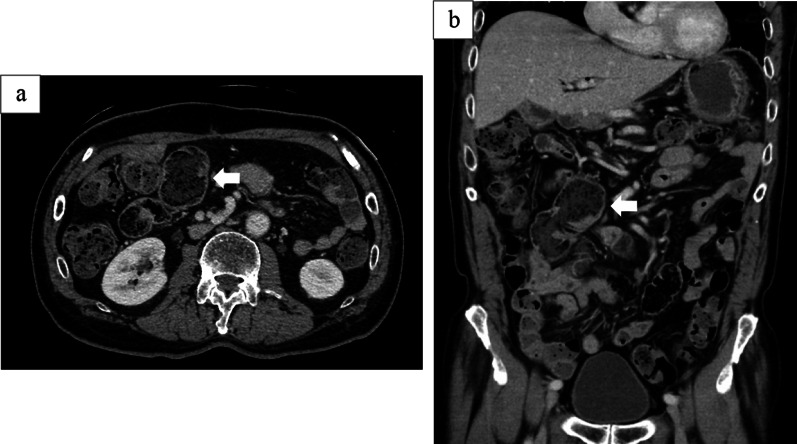
CT imaging showed dilatation of the Roux-Y-limb (white arrow)

**Fig. 3:**
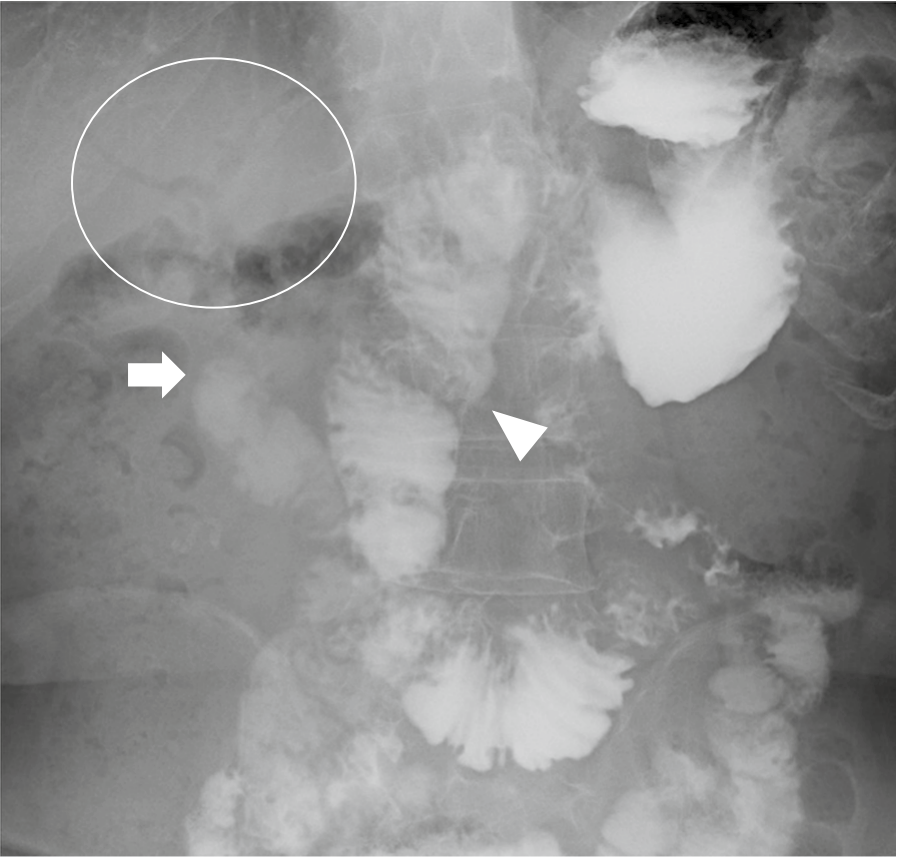
Oral gastrografin contrast examination showed dilated Roux-Y-limb (arrowhead) and regurgitation of the contrast medium toward the hepaticojejunostomy (white arrow) and pneumobilia (circle)

Since stenosis at the hepaticojejunostomy was suspected as the cause of repeated cholangitis, a small bowel endoscopy was performed. Fluoroscopic endoscopic findings showed no stenosis at the anastomosis and no stones in the intrahepatic bile duct (Fig. 1a, b). Small bowel endoscopy also confirmed that there was no obvious stenosis or obstruction in the gastrointestinal tract, including the afferent loop. Upon reviewing the CT scan at the onset of cholangitis, the afferent loop was noticeably dilated (Fig. 2a, b), and stasis and reflux in the same area were suspected of causing cholangitis. Therefore, gastrointestinal imaging was performed to confirm reflux. Gastrointestinal imaging revealed the presence of regurgitation, with findings that the orally ingested contrast agent passed through the afferent loop and then flowed back through the hepaticojejunostomy (Fig. 3). The patient was diagnosed with reflux cholangitis due to chronic reflux into the afferent loop after oral intake.

**Fig. 4:**
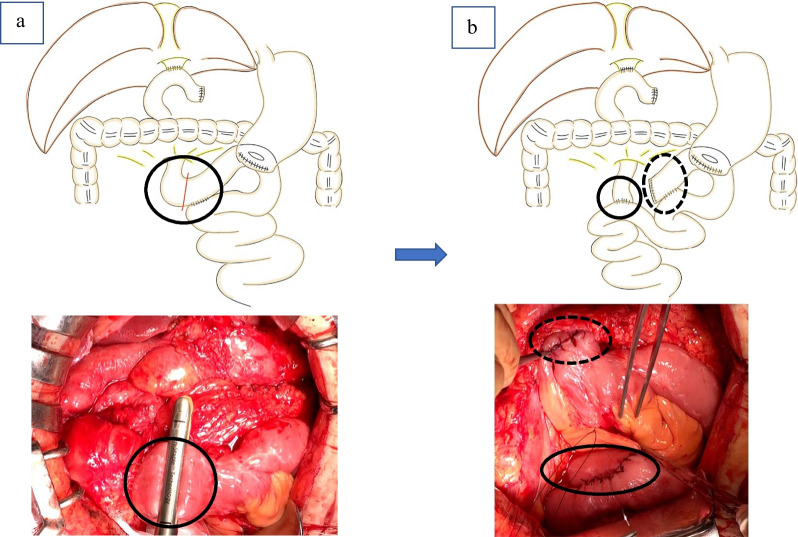
Tract conversion surgery [Billroth II (**a**) to Roux-en-Y type (**b**)]

Various treatment options were considered, and the decision was made to perform tract conversion surgery (from the Billroth II method to the Roux-en-Y type technique) as a radical treatment based on previous reports [5] for the purpose of separating the gastrojejunostomy from the biliary tract. The patient was fully informed and consented to the procedure. The abdomen was opened through a median upper abdominal incision. As in the preoperative imaging studies, a dilated, elevated bowel was seen at the afferent loop. The afferent loop was separated with a stapler on the side of the hepaticojejunostomy near the Braun anastomosis. To prevent reflux, tract conversion was performed by refashioning the jejunojejunostomy downstream (Fig. 4a, b). The operation time was 1 h, and bleeding was minimal. Drinking water was started on postoperative day 1, and oral intake was started on postoperative day 3. The patient did not develop a fever after oral intake and was discharged from the hospital on the 8th day. Four years after the second surgery and 5 years after the first surgery, the patient is now cholangitis-free, and cancer has not recurred.

## Discussion

Cholangitis after biliary reconstruction is a relatively common complication encountered in hepatobiliary pancreatic surgery. Furthermore, with improved surgical outcomes, there are more opportunities to address cholangitis, which is a late complication. Several reports have been made regarding the risk of developing postoperative cholangitis. Some reports indicate that the risk of cholangitis after biliary reconstruction is affected by benign diseases, an extended operating time, elevated CRP levels, elevated ALP levels, chemotherapy, recurrence, male sex and the presence of postoperative complications [2, 3, 8], while others report that no specific cause can be identified [7, 9]. Regarding the time of onset, postoperative cholangitis has been reported to occur within 2 months to 82 months [7], but most cases occur within 2 years [8, 9]. Regarding the frequency of cholangitis, one report revealed a frequency of more than 10 times, as in the present case, in approximately one-quarter of cases [8]. Although postoperative cholangitis has been reported as described above, there are several issues that need to be addressed. First, the definition and severity of postoperative cholangitis were unclear in cases reported prior to the development of the TG13 guidelines [10]. Because there is now a uniform standard, postoperative cholangitis should be validated based on the definition of the TG18 guidelines in the future [11]. Second, there is a lack of uniformity in terminology as well as in the pathogenesis of the disease. The fact that the same condition has been reported under different names, such as “afferent loop syndrome,” “sump syndrome,” and “postoperative cholangitis,” is also problematic [12, 13]. Since this condition is frequently encountered in the postoperative period, it is desirable to have a unified terminology and definition of the condition in the future. Third, most reported cases of postoperative cholangitis are discussed together with and without stenosis. Although cholangitis can occur in both cases, the pathophysiology and treatment are different, so the discussion should be separate. In clinical practice, it is important to evaluate patients with postoperative cholangitis for the presence of obstruction. The diagnosis is relatively easy in the presence of obstruction, such as anastomotic stenosis or stones in the bile duct. In some cases, stones in the Roux-en-Y intestinal tract have been reported, resulting in cholangitis [14]. Endoscopic, percutaneous, or surgical treatment is indicated for each cause, and symptoms improve when the obstruction is removed. On the other hand, in most cases, cholangitis is diagnosed clinically when fever, abdominal pain, and elevated hepatobiliary enzyme levels are observed without any other apparent cause. Most cases are treated with antimicrobial agents, because the symptoms are mild and not frequently recurrent, so the search for the cause of the disease does not extend to the treatment of the disease in most cases. However, it is also true that there are patients, such as the present patient, in whom symptoms recur frequently and quality of life declines markedly. In a recurring case, such as the present case, it is essential to exclude obstruction and confirm the presence of reflux and stasis by imaging studies to determine a treatment plan. The pathophysiology of postoperative nonobstructive recurrent cholangitis reportedly has two causes [14]: a mechanical effect due to adhesions and flexion of the afferent loop, and a functional effect due to dysmotility of the vagal denervation or proximal disconnection of the afferent loop from the main pacemaker in the duodenum. No mechanical cause was observed in the present case, so we assumed a functional cause. Endoscopy is useful in excluding obstruction, and if stenosis is detected, it is also useful in diagnosing benign or malignant conditions and treating the obstruction at the same time. Percutaneous transhepatic cholangiography, MRCP, and DIC–CT may also be useful in assessing the presence of stenosis. The presence of orally administered gastrografin in the afferent loop or biliary tree is the most standard evaluation of reflux and stagnation. Some reports have reported that scintigrams might also be useful [13]. The above tests should be used to determine the indication for surgery. Some studies have suggested that a longer afferent loop or Roux-en-Y limb is a factor that prevents reflux and stasis in patients with biliary reconstruction [15, 16], but there are also reports that shorter limb lengths are associated with fewer complications, so a certain view has not been reached [17]. In this case, the indication for surgery was also determined after careful consideration, referring to previous cases in which surgery was performed for nonobstructive retrograde cholangitis that developed after biliary reconstruction surgery [4, 5, 18, 19]. The procedure was determined with reference to the Roux-en-Y extension to increase the distance between the route of oral intake and the bile duct jejunal anastomosis, among others [5]. Other reports have described cases in which a valve was created to prevent reflux [4] or the reconstructive configuration was changed to prevent reflux [18, 19], with good results. It is important to consider effective surgical techniques that are appropriate for the cause of the reflux. The indication and timing of surgery for postoperative nonobstructive recurrent cholangitis should take into account the severity of the patient’s condition and the appropriateness of the procedure. Since many cases can be treated conservatively, surgery should be considered for severe symptoms, such as liver abscess, sepsis, or when symptoms are frequent and cause a significant decrease in quality of life, after confirming that reflux is clearly present. It is important to fully consider each case individually. In addition, recent advances in endoscopic treatment have led to reports of the effectiveness of stenting with antireflux valves, which is considered less invasive [20]. This is a promising, minimally invasive treatment for patients who have recurrent postoperative cholangitis but cannot tolerate surgery.

## Conclusion

We experienced a case of a patient with repeated nonobstructive cholangitis who was successfully treated with surgery. In the treatment of repeated postoperative nonobstructive cholangitis, surgery has been successful in some cases and is worth considering as a treatment option after thorough evaluation.

## Data Availability

The authors declare that all the data are available within this article.

## Abbreviations

CT: Computed tomography
CRP: C-reactive protein
ALP: Alkaline phosphatase
TG: Tokyo guideline
MRCP: Magnetic resonance cholangiopancreatography
DIC: Drip infusion cholecystocholangiography
